# Ilioinguinal-Iliohypogastric Block for Open Inguinal Hernia Repair: A Prospective Observational Study

**DOI:** 10.4274/TJAR.2026.252356

**Published:** 2026-08-28

**Authors:** Silvia De Pinto, Alessandro De Cassai, Margherita Iuzzolino, Giovanni Zecchino, Annalisa Boscolo, Giacomo Sarzo, Burhan Dost, Giuseppe Franco, Marina Munari

**Affiliations:** 1 University Hospital of Padua, Department of Anaesthesia, Intensive Care and Neurointensive Care, Padua, Italy; 2 University of Padua, Department of Medicine DIMED, Padua, Italy; 3 University Hospital of Padua, Department of Anaesthesia and Intensive Care, Padua, Italy; 4 University of Padua, Department of Cardiac, Thoracic, Vascular Sciences, and Public Health, Padua, Italy; 5 Sant' Antonio Hospital, Clinic of Surgery, Padua, Italy; 6 Ondokuz Mayıs University School of Medicine, Department of Anaesthesiology and Reanimation, Samsun, Türkiye; 7 University of Padua Faculty of Medicine and Surgery, Padua, Italy

**Keywords:** Ambulatory surgery, block failure predictors, ilioinguinal-iliohypogastric nerve block, inguinal hernia repair, regional anaesthesia, ultrasound-guided nerve block

## Abstract

**Objective:**

This study aimed to evaluate the effectiveness of the ultrasound-guided ilioinguinal-iliohypogastric (II-IH) nerve block under mild intravenous sedation as a sole anaesthetic technique for open inguinal hernia repair and to identify factors independently associated with clinical inadequacy. The central research question was whether the II-IH block alone, when combined with intravenous sedation, could provide adequate anaesthesia for this procedure in a day-case setting.

**Methods:**

In this prospective observational study conducted at a university hospital, 342 adult patients undergoing unilateral open inguinal hernia repair with II-IH block were enrolled. The primary outcome was a composite of the need for intraoperative opioid supplementation, additional local anaesthetic, or conversion to general anaesthesia. Logistic regression was used to assess predefined predictors of clinical inadequacy.

**Results:**

Out of 342 patients, 233 (68.2%) successfully completed surgery with an II-IH block and mild intravenous sedation as the sole anaesthetic technique. In the remaining 109 patients (31.8%), only 9 (2.6%) required conversion to general anaesthesia. Logistic regression identified anaesthetic volume—but not dose—as significantly associated with clinical inadequacy (*P* < 0.001).

**Conclusion:**

The II-IH block, when combined with sedation, is a feasible and effective anaesthetic approach for a substantial proportion of appropriately selected patients undergoing open inguinal hernia repair. This approach may represent a practical alternative to spinal or general anaesthesia.

**Main Points**

• Ultrasound-guided ilioinguinal-iliohypogastric (II-IH) nerve block combined with intravenous sedation provided adequate anaesthesia as the sole anaesthetic technique in more than two-thirds (68.2%) of patients undergoing open inguinal hernia repair and was associated with a low conversion rate to general anaesthesia (2.6%).

• In cases of clinical inadequacy, they were most often managed with minimal supplementation (additional local anaesthetic or opioids), supporting the feasibility of this approach in a day-case surgical setting.

• The volume of local anaesthetic—but not the total dose—was independently associated with clinical inadequacy, suggesting that excessive volume may reduce block effectiveness when the concentration of the local anaesthetic is lowered.

• Ultrasound-guided II-IH block may be an alternative to general or spinal anaesthesia for open inguinal hernia repair, but optimizing anaesthetic volume and sedation strategy is crucial to maximize procedural success and patient safety.

## Introduction

Inguinal hernia repair surgery is one of the most commonly performed surgeries worldwide.^1^ This operation can be conducted under regional anaesthesia (namely spinal anaesthesia, epidural anaesthesia, nerve block or local anaesthesia) or general anaesthesia and the choice between these options largely depends on anaesthesiologist and surgeon habits, preference and expectations of the patients, reliability of the method, and ease and cost of the application.^2^

However, general anaesthesia and neuraxial anaesthesia options may not be the most appropriate choices for a surgery usually performed in a day-case setting due to possible consequences of these techniques as undesirable haemodynamic responses, urinary retention, postdural puncture headache, prolonged recovery and hospital stay.^3^ Moreover, for this surgery patients undergoing neuraxial anaesthesia or general anaesthesia express a lower satisfaction than patients receiving other anaesthesia options.^3^

The ilioinguinal-iliohypogastric (II-IH) nerve block is a regional anaesthetic technique that targets the nerves arising from the L1 spinal nerve root. These nerves course between the internal oblique and transversus abdominis muscles, and can usually be visualized and blocked under ultrasound guidance near the anterior superior iliac spine. The ultrasound-guided II-IH block has been used in hernia surgery, either alongside general anaesthesia to reduce opioid and analgesic use,^4^ or as a standalone technique with mild sedation.^5^ However, its success rate as a sole anaesthetic for open hernia repair remains unclear, and predictors of clinical inadequacy are not well established.

The primary aim of this study was to assess the efficacy of the II-IH block as a standalone technique for open inguinal hernia repair. A secondary aim was to identify factors independently associated with the primary outcome.

## Methods

This prospective observational study was conducted in the operating room of Padua University Hospital, Padua, Italy. Approved by the Clinical Research Ethics Committee of Azienda Ospedale University Hospital (approval no: 5804/AO/23, date: 05.09.2023). Registered prospectively on ClinicalTrials.gov (NCT06121726).

Patients aged 18 years or older undergoing unilateral open inguinal hernia repair with II-IH block as the main anaesthesia technique were evaluated for inclusion. Exclusion criteria included a documented allergy to local anaesthetics; cardiac, renal, or hepatic failure; central or peripheral neuropathies; abnormal coagulation tests; infection at the surgical site; and inability to understand or provide written informed consent, which was a mandatory requirement for participation in this study.

Given the observational nature of the study, participation did not alter standard patient management. Therefore, only a brief description of routine care at our hospital is provided in this manuscript. Patients are typically monitored using electrocardiography, pulse oximetry, and non-invasive blood pressure measurement. Following the establishment of peripheral venous access and premedication with midazolam (1-2 mg) and fentanyl 100 µg according to institutional protocol, the II-IH block is performed using ropivacaine, a long-acting local anaesthetic, under ultrasound guidance after proper skin disinfection. At our institution, volumes ranging from 4 to 20 mL and concentrations between 0.4% and 1% are typically used; however, the final dose and concentration are determined at the discretion of the attending anaesthesiologist. The II-IH nerves can be visualized in the transversus abdominis plane (TAP) using a high-frequency linear probe placed on the iliac crest and oriented obliquely between the iliac crest and the umbilicus. If the nerves are not visualizable despite accurate ultrasound scanning, the local anaesthetic is injected into the TAP in close proximity to the iliac crest. Mild sedation was performed with a target controlled infusion pump using the Eleved model;^6^ the target concentration was determined at the discretion of the attending anaesthesiologist, typically guided by depth-of-anaesthesia monitoring using the SedLine® system (Masimo, Irvine, CA, USA), with the aim of achieving values consistent with mild sedation (Patient State Index target range: 50-70). All the anaesthesiologists performing the blocks had five or more years of experience in regional anaesthesia.

We collected the following patient-related variables: age (years), gender (male/female), weight (kg), height (cm), American Society of Anesthesiologists physical status (ASA-PS), ultrasound visualization of the II-IH nerves (yes/no), volume of local anaesthetic administered (mL), total dose of ropivacaine (mg), time from block to surgical incision (minutes), dose of propofol used (expressed as effect-site concentration, µg kg^-1^), and any complications.

The time from block to surgical incision was defined as the interval between completion of the local anaesthetic injection and the skin incision. This variable was recorded to assess whether a shorter interval might be associated with incomplete block onset. The timing primarily depended on surgical workflow and was not modified by the attending anaesthesiologist for the purposes of this study.

We hypothesize that II-IH, when combined with mild intravenous sedation, can be used as the sole anaesthetic technique for open inguinal hernia surgery. For this reason the primary outcome was a composite endpoint defined as the occurrence of any of the following during surgery due to inadequate anaesthetic plan as determined by the attending anaesthesiologist: (a) administration of additional local anaesthetic, (b) intraoperative opioid use, or (c) conversion to general anaesthesia. Pre-specified predictors of clinical inadequacy, which were defined and registered prior to the study, included age, body mass index, time from block to incision, local anaesthetic dose, local anaesthetic volume, and ultrasound visualization of the nerves.

As a secondary post hoc outcome, we evaluated the incidence of conversion to general anaesthesia.

A large language model (ChatGPT 5.2; OpenAI, San Francisco, CA, USA) was used solely to review and improve the manuscript’s grammar and fluency. All content was critically reviewed and verified by the authors, who retain full responsibility and accountability for the final version of the manuscript.^7^

### Statistical Analysis

Due to the observational nature of the study and the absence of prior data, a formal sample size calculation was not performed. Instead, we aimed to enroll as many patients as possible over a two-year period, up to a maximum of 400 patients, as approved by the Institutional Review Board.

The Shapiro-Wilk test was used to assess the normality of variable distributions. Continuous variables with normal distribution are presented as mean ± standard deviation, while non-normally distributed variables are reported as median with interquartile range (Q1, Q3). Categorical variables are expressed as absolute numbers and percentages.

Comparisons between groups were conducted using the Student’s t-test for normally distributed continuous variables, the Mann-Whitney U test for non-normally distributed continuous variables, and the chi-square test or Fisher’s exact test for categorical variables, as appropriate.

To identify predictors of the main outcome, a logistic regression analysis was performed, with the composite main outcome as the dependent binary variable and the pre-specified predictors as independent variables. Results are reported as odds ratios (OR) with 95% confidence intervals (CI).

Statistical significance was defined as a two-tailed *P *value < 0.05. All analyses were conducted using R software, version 4.0.2 (the R foundation for statistical computing).

## Results

**Figure 1:**
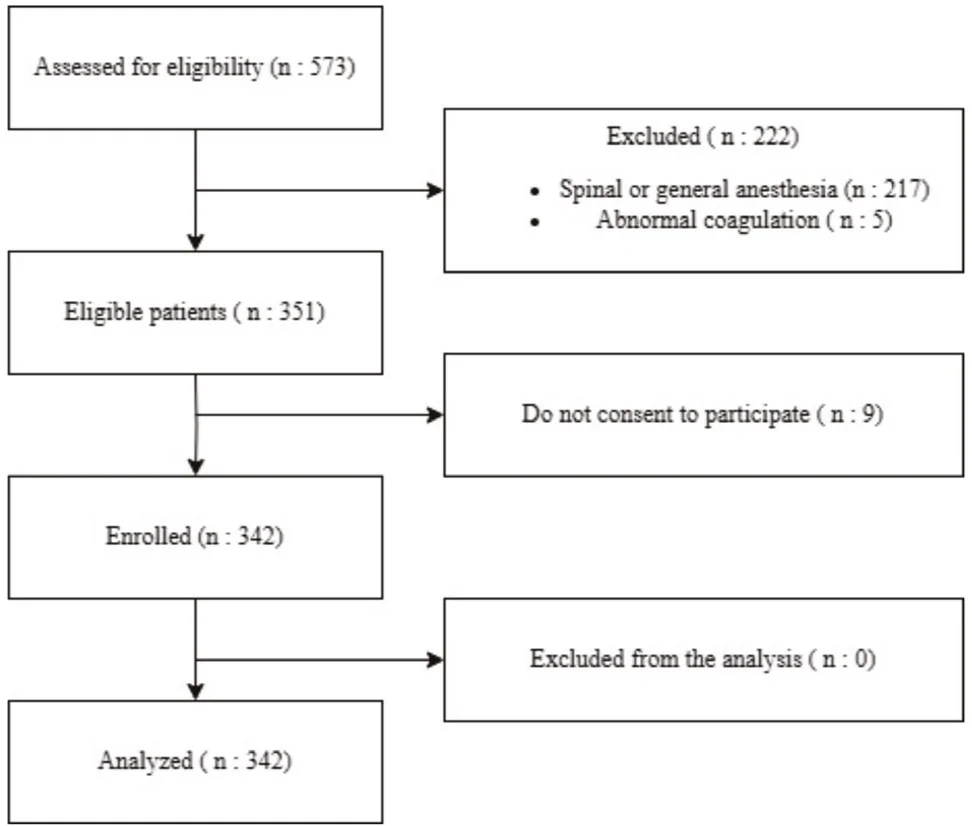
Enrollment flow-chart.

A total of 573 patients were evaluated for potential inclusion between October 13, 2023, and May 2, 2025. Of these, 236 patients were excluded: 216 because the attending anaesthesiologist selected an alternative anaesthetic technique (either general or spinal anaesthesia); 5 because they had abnormal coagulation tests; and 9 because they did not provide informed consent. This left 342 patients enrolled in the study (Figure 1). Patient characteristics for both groups—those with successful and failed blocks—are summarized in Table 1.

Among the included patients, 233 (68.2%) successfully underwent open inguinal hernia repair with an II-IH nerve block and mild intravenous sedation as the sole anaesthetic technique. The remaining 109 patients (31.8%) required additional local anaesthesia, administration of opioids, or conversion to general anaesthesia.

In particular, 85 patients required supplementary local anaesthetic administered by the surgeon; 36 received an additional 100 µg dose of fentanyl (interquartile range: 100-100); and nine patients (2.6%) required conversion to general anaesthesia. Of those converted, eight cases were attributable to inadequate anaesthesia despite the administration of both local anaesthetic and opioids, while one conversion was necessitated by intraoperative bronchospasm. Additionally, in five patients (1.5%), all of whom were in the success group, a femoral nerve block complicated the postoperative period, in all of these patients 20 mL of local anaesthetic were used. This complication necessitated overnight observation, resulting in a one-day prolongation of hospitalization. All affected patients had complete sensory and motor recovery by the first postoperative day and were discharged without further complications.

**Figure 2:**
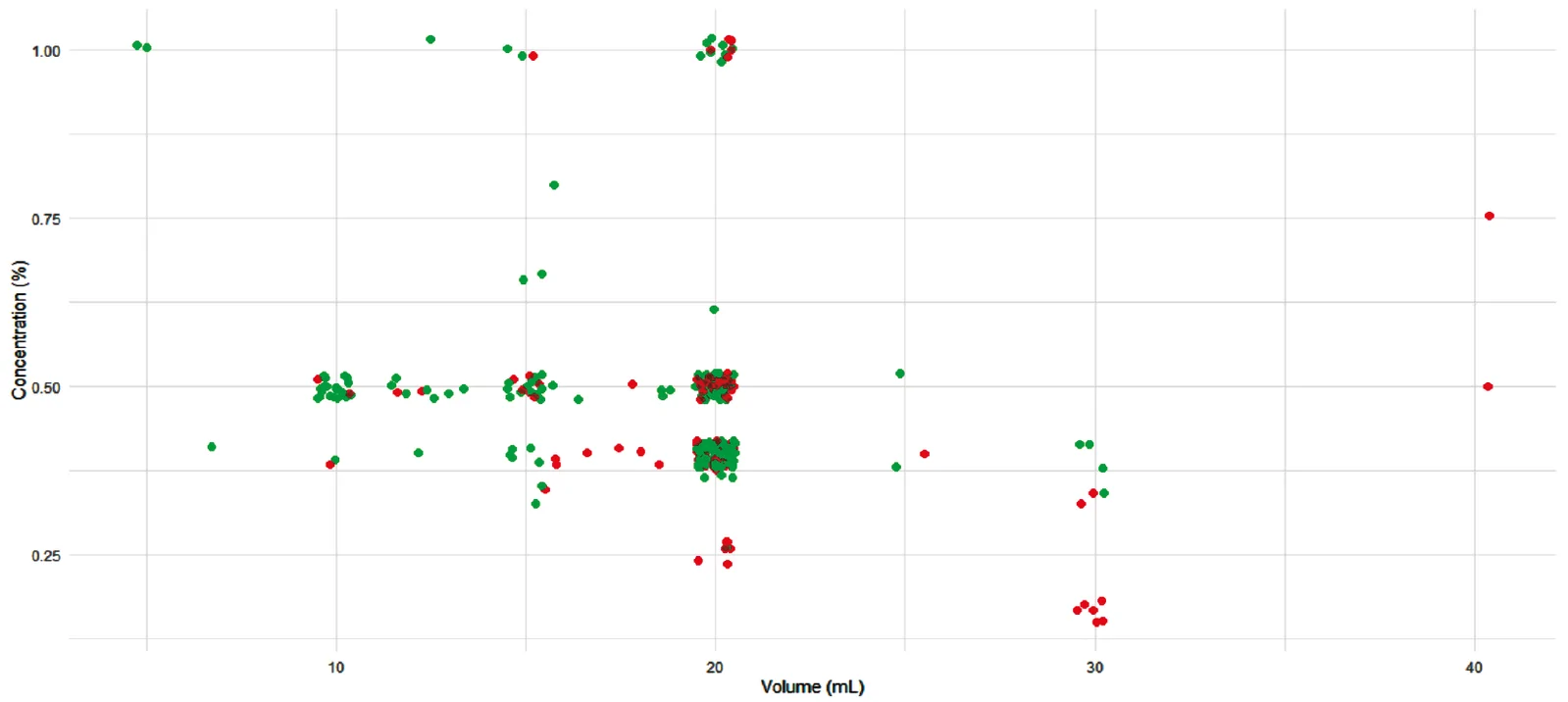
Scatter plot showing the association between injected volume and ropivacaine concentration, stratified by block outcome (failure in red, success in green). Each point corresponds to one patient. To minimize overplotting due to the limited number of concentration levels, a small vertical jitter was applied (±0.02%). Consequently, some points may appear marginally outside the true concentration values for visualization purposes only.

A scatter plot displaying the relationship among block clinical adequacy, volume, and concentration is shown in Figure 2.

In the logistic regression of the pre-specified predictors, only the volume—but not the dose—of local anaesthetic was statistically significant (*P* < 0.001), as shown in Table 2. Although the level of propofol sedation reached statistical significance in univariable analysis, it was not included in the multivariable logistic regression model for two reasons. First, according to our predefined statistical analysis plan, only variables identified a priori as clinically relevant were entered into the model. Second, the level of intraoperative propofol sedation may represent a consequence rather than an independent predictor of block inadequacy, since patients with insufficient block efficacy are more likely to receive higher levels of sedation. Including this variable in the model could, therefore, introduce reverse causality and bias the estimates. Table 3 compares patients who required conversion to general anaesthesia with those who did not.

## Discussion

The main finding of this study is that open inguinal hernia repair can be effectively performed using the II-IH nerve block under mild intravenous sedation as the sole anaesthetic technique in the majority of cases (68.2%). This finding suggests that the II-IH nerve block may be considered for selected patients undergoing inguinal hernia repair.

Even among the remaining 31.8% of patients who needed further intervention to achieve an adequate anaesthetic plane, most were managed with minimal supplementation, either with local anaesthetics or opioids. The rate of conversion to general anaesthesia remained low (2.6%), which further reinforces the clinical feasibility of this technique, especially in the context of ambulatory surgery. However, femoral nerve block occurred in 1.5% of the overall population, likely due to the anatomical proximity between the fascia iliaca compartment and the TAP.^8^ This potential complication should always be considered, as it may have disproportionate implications for ambulatory surgery, where early mobilization and timely discharge are essential.

Pain during open inguinal hernia repair is not exclusively mediated by the II-IH nerves. The genital branch of the genitofemoral nerve may also contribute significantly to perioperative pain due to its anatomical course within the inguinal canal and its close association with the spermatic cord, although its trajectory and sensory distribution demonstrate considerable interindividual variability.^9, 10^ Surgical manipulation, hernia sac dissection, and mesh placement may stimulate this nerve, potentially leading to incomplete analgesia. Consequently, insufficient blockade of the genitofemoral nerve may partly account for cases of inadequate anaesthesia despite an apparently successful II-IH block; and the block of all of these nerve blocks have reported higher patient satisfaction than the II-IH alone.^11^ However, a previous study compared the combined II-IH and genitofemoral nerve block with spinal anaesthesia in a similar surgical population. Although the authors reported reduced postoperative pain in patients receiving the II-IH block compared with spinal anaesthesia, 13.3% of patients in the II-IH group experienced block failure and were excluded from the final analysis. Moreover, the criteria used to define block failure were not clearly specified, which limits comparability with our reported failure rates.^12^

II-IH block has long been established as a preferred regional anaesthetic technique for pain control after open inguinal hernia repair. Its effectiveness has been shown to be superior to that of the TAP involve injections into a similar fascial plane, the II-IH block allows more targeted nerve blockade.^13^

However, this superiority has been demonstrated only when the II-IH block is performed under ultrasound guidance. When performed using a landmark-based (blind) technique, the II-IH block has been shown to be inferior to an ultrasound-guided TAP block.^14^

Although optimal nerve visualization under ultrasound was numerically higher in the failure group, this difference did not reach statistical significance. The lack of statistical significance suggests that the observed difference may be attributable to random variation rather than to a true underlying association, i.e., it likely reflects chance alone.

The II-IH block has been proposed for inguinal hernia repair because it offers several advantages over general and spinal anaesthesia. These include reduced opioid consumption,^4, 15^ higher satisfaction,^16^ potential reductions in chronic postoperative pain—though findings remain conflicting—^1, 17^ and earlier hospital discharge.^15, 18^

Of particular interest in our study is the relationship between clinical inadequacy and anaesthetic volume. While our institution has traditionally employed relatively large volumes, our results suggest that larger volumes may not confer additional benefit and may, in fact, be counterproductive. When the total dose of anaesthetic is held constant, increasing volume necessarily decreases concentration, which could reduce the effectiveness of the block as a sole anaesthetic technique. Furthermore, variations in injectate volume may influence the spread within fascial planaes, alter local anaesthetic distribution, and increase the likelihood of unintended blockade of adjacent nerves. Notably, femoral nerve block occurred in a subset of patients and may have been facilitated by greater spread of injectate associated with larger volumes. Moreover, the existing literature indicates that the minimum effective volume for successful II-IH block is approximately 0.9 mL per nerve, and even with such small volumes, selective blockade of the ilioinguinal or of the iliohypogastric nerves individually is not reliably achievable suggesting that even with small volumes both nerves are blocked.^19, 20^ This suggests that precision in volume and concentration—rather than simply increasing total volume—may be more critical to the success and safety of the block.

An important consideration is the anatomical and surgical complexity of the inguinal region in open hernia repair. The II-IH block primarily targets the anterior branches of T12-L1; however, the surgical steps involved—including skin incision, spermatic cord manipulation, deep dissection, and mesh placement—may recruit nociceptive input beyond this territory. Specifically, the lateral cutaneous branches of T12-L1 and, critically, the genital branch of the genitofemoral nerve, which travels through the inguinal canal in close proximity to the spermatic cord, may contribute significantly to intraoperative nociception, particularly during cord manipulation and deep dissection. It follows that a subset of cases requiring analgesic supplementation may reflect incomplete anatomical coverage rather than a true technical failure of the block-a distinction with relevant clinical and methodological implications.

In this regard, our study should not be interpreted as an investigation of the practitioner’s ability to successfully perform the II-IH block, but rather as an assessment of the extent to which the II-IH block provides adequate sensory coverage of the surgical field during open inguinal hernia repair. This distinction, while subtle, is substantial. Our findings indicate that the proportion of cases in which the II-IH block alone achieves complete intraoperative analgesia is appreciable but not absolute, suggesting that the anatomical territory of this block may be insufficient to cover the full nociceptive input of the surgical field in a non-negligible subset of patients.

For this reason, practitioners may consider adding complementary fascial-plane blocks to broaden sensory coverage of the inguinal region. The lateral and posterior TAP blocks may provide additional coverage of the lateral cutaneous contributions from T12-L1, while the transversalis fascia plane block has emerged as a promising approach to target the genitofemoral nerve genital branch within the preperitoneal space^21^-a territory not reliably reached by the II-IH block alone. Whether these complementary blocks should be systematically combined with the II-IH block or selectively employed based on intraoperative analgesic requirements remains an open and clinically relevant question. Prospective studies comparing the analgesic efficacy of the II-IH block alone versus that of combined fascial plane block strategies are warranted to optimize regional anaesthesia protocols for open inguinal hernia repair.

### Study Limitations

Our study has several limitations that warrant consideration. First, its single-center observational design may limit the generalizability of the findings to other clinical settings. In addition, the lack of standardization of drug concentrations used for both the regional block and sedation and the absence of intraoperative data on pain or hemodynamics may have introduced confounding factors, thereby affecting the consistency of the results. Multicenter prospective studies with standardized protocols are needed to validate and expand upon these findings. Moreover, the absence of a control or comparison group represents an important methodological limitation. As a result, the findings reflect only observational outcomes, and no conclusions can be drawn regarding causality or the superiority or inferiority of the II-IH technique compared with other regional anaesthesia approaches.

Second, the study is subject to selection bias. Of the 573 patients who underwent open inguinal hernia repair at our institution during the study period, 231 were excluded, primarily because the attending anaesthesiologist chose an alternative anaesthetic technique. Unfortunately, we did not collect detailed data on the excluded patients, but it is reasonable to hypothesize that the anaesthetic approach may have been influenced by a variety of factors, including individual preferences of the anaesthesiologist, surgeon, or patient; hernia size; anticipated technical difficulty of the procedure; or the patient’s underlying comorbidities. Consequently, more complex or higher-risk cases may have been disproportionately excluded, potentially limiting the generalizability of our findings. However, this type of bias is almost unavoidable in an observational study, as the conduct of the attending anaesthesiologist could not be modified by the researchers. For this reason, prospective and multicenter studies are deemed necessary, and while our findings offer an interesting perspective on the use of II-IH nerve block under mild intravenous sedation as a sole anaesthetic technique for inguinal hernia repair, they should be interpreted with caution.

Third, in addition to anaesthetic factors, surgical variables may have influenced block adequacy. The extent of surgical dissection can vary considerably depending on hernia characteristics, including size and complexity, incision length, degree of spermatic cord mobilization, and mesh size and plane of placement. More extensive tissue manipulation may increase nociceptive input from areas not consistently covered by an ilioinguinal-iliohypogastric block alone, potentially contributing to the inadequacy of the II-IH technique. Because these surgical factors were not systematically recorded in the present study, their impact on block success could not be evaluated. This inability to evaluate their impact should be considered a limitation of our analysis.

No a priori sample size calculation or power analysis was performed. Given the number of variables included in the logistic regression model, the study may have been underpowered to detect smaller but clinically relevant associations; consequently, the stability of the multivariable estimates should be interpreted with caution.

## Conclusion

Our findings suggest that ultrasound-guided II-IH nerve block, combined with intravenous sedation, can provide adequate anaesthesia for open inguinal hernia repair in a substantial proportion of appropriately selected patients. This approach may represent a practical alternative to spinal or general anaesthesia, reducing exposure to more invasive anaesthetic techniques while maintaining satisfactory surgical conditions.

## Ethics

**Ethics Committee Approval:** Approved by the Clinical Research Ethics Committee of Azienda Ospedale University Hospital (approval no: 5804/AO/23, date: 05.09.2023).

**Informed Consent:** Written informed consent was obtained from all participants included in the study.

## Figures and Tables

**Table 1. Characteristics of Study Participants:** 

**Success**	**Yes (233)**	**No (109)**	***P* value**
Gender (M)	210 (90.1%)	101 (92.7%)	0.447
Age (years)	60.18±14.64	60.77±12.26	0.694
ASA-PS	2 (1-2)	2 (1-2)	0.661
BMI (kg m^2-1^)	24.61±2.83	24.95±3.01	0.338
US optimal visualization (yes)	155 (66.5%)	80 (73.4%)	0.201
LA dose (mg)	80 (80-100)	80 (75-100)	0.978
LA volume (mL)	20 (15-20)	20 (20-20)	0.002*
Time to incision (min)	18.35±9.88	17.67±10.79	0.576
Propofol (cet)	1.5 (1.2-2.0)	1.8 (1.3-2.2)	0.009*

*: Statistically significant at *P* value < 0.050

ASA-PS, American Society of Anesthesiologists physical status; BMI, body mass index; Cet, target concentration at effect site; LA, local anaesthetic; M, male; US, ultrasound

**Table 2. Logistic Regression (Binary Composite Main Outcome as Dependent Variable):** 

**Variable**	**OR (95% CI)**	***P* value**
Age (years)	1.01 (0.99- 1.03)	0.457
LA dose (mg)	1.00 (1.00-1.01)	0.388
LA volume (mL)	1.13 (1.06- 1.21)	<0.001*
Time to incision (min)	0.99 (0.96-1.01)	0.362
US optimal visualization (yes)	1.58 (0.93- 2.74)	0.098
BMI (kg m^2-1^)	1.04 (0.96- 1.13)	0.379

*: Statistically significant at *P* value < 0.050

BMI, body mass index; LA, local anaesthetic; US, ultrasound; OR, odds ratio; CI, confidence interval

**Table 3. Group Characteristics for the Post-hoc Analysis Evaluating Conversion to General Anaesthesia:** 

**Conversion to GA**	**No (333)**	**Yes (9)**
Gender (M)	303 (91.0%)	8 (88.9%)
Age (years)	60 (51-70.25)	66 (56-68)
ASA-PS	2 (1-2)	2 (1-2)
BMI (kg m^2-1^)	24.45 (22.86-26.57)	23.53 (22.78-26.99)
US optimal visualization (yes)	231 (69.4%)	4 (44.4%)
LA dose (mg)	80 (75-100)	80 (75-80)
LA volume (mL)	20 (20-20)	20 (15-20)
Time to incision (min)	15 (10-25)	15 (10-20)
Propofol (cet)	1.5 (1.2-2)	2 (1.8-3.5)

Given the exploratory nature of the analysis, we chose not to perform formal statistical comparisons between groups

ASA-PS, American Society of Anesthesiologists physical status; BMI, body mass index; Cet, target concentration at effect site; GA, general anaesthesia; LA, local anaesthetic; M, male; US, ultrasound
